# Approximating Optimal Behavioural Strategies Down to Rules-of-Thumb: Energy Reserve Changes in Pairs of Social Foragers

**DOI:** 10.1371/journal.pone.0022104

**Published:** 2011-07-12

**Authors:** Sean A. Rands

**Affiliations:** Centre for Behavioural Biology, University of Bristol, School of Veterinary Science, Langford, Bristol, United Kingdom; University of Chicago, United States of America

## Abstract

Functional explanations of behaviour often propose optimal strategies for organisms to follow. These ‘best’ strategies could be difficult to perform given biological constraints such as neural architecture and physiological constraints. Instead, simple heuristics or ‘rules-of-thumb’ that approximate these optimal strategies may instead be performed. From a modelling perspective, rules-of-thumb are also useful tools for considering how group behaviour is shaped by the behaviours of individuals. Using simple rules-of-thumb reduces the complexity of these models, but care needs to be taken to use rules that are biologically relevant. Here, we investigate the similarity between the outputs of a two-player dynamic foraging game (which generated optimal but complex solutions) and a computational simulation of the behaviours of the two members of a foraging pair, who instead followed a rule-of-thumb approximation of the game's output. The original game generated complex results, and we demonstrate here that the simulations following the much-simplified rules-of-thumb also generate complex results, suggesting that the rule-of-thumb was sufficient to make some of the model outcomes unpredictable. There was some agreement between both modelling techniques, but some differences arose – particularly when pair members were not identical in how they gained and lost energy. We argue that exploring how rules-of-thumb perform in comparison to their optimal counterparts is an important exercise for biologically validating the output of agent-based models of group behaviour.

## Introduction

Animals can gain many benefits from living in groups [1], [2], such as an enhanced ability to find food [3], [4], avoid predation [5]–[8], or make accurate decisions [9]–[13]. However, group living brings costs too, such as enhancing the risks of parasitism and infection [14], [15], increasing competition for resources [4], or increasing detectability of the group to predators [16], [17]. In order to understand why individuals of a particular species behave the way they do when they are in a group involves understanding how these costs and benefits are traded off against each other. If we are interested in exploring the group behaviour of species using a modelling framework, we therefore need to be careful to reflect those trade-offs that are the most important within the model.

Models of social behaviour typically attempt either to identify behaviours for a given set of environmental or social conditions, or else simulate the behaviour of the individuals within the group to explore how the group behaviour changes in response to parameter manipulation. In the former approach, it is assumed that the behaviours that will be shown by individuals are optimal (maximising some measure of fitness), by considering these behaviours from a functional perspective [18]–[20]. In contrast, a simulation approach may incorporate an individual's presumed rule-set within a group simulation, and explore how changing these rule-sets affect the behaviour of the group (*e.g.* [13], [21]–[25], where the rules used can come from careful characterisation of biological systems, *e.g.* [26]–[29]). These two approaches explore social behaviour from two different perspectives. The former approach explores social behaviour from one direction, where the best behaviour of an individual is calculated in response to the environment and the group. The latter explores the phenomenon from the opposite direction, and considers how individual behaviours lead to behaviours at the level of the group. Although complementary, many studies focus solely on one or other of these approaches. Using the two together may lead to a deeper understanding of a given system's behaviour and evolution [30], [31].

In a series of models examining social foraging behaviour that combined both approaches, Rands *et al*. firstly constructed a dynamic game which identified optimal rule-sets for a pair of foragers, based upon a trade-off between energetic intake and predation risk when foraging alone or together [30], [32]. Simplified variations on the optimal rule-sets from these models were then adapted to be used within individual-based simulations exploring group behaviour [33], [34]. Simplified ‘rule-of-thumb’ versions of the rules were necessary, not only because the original optimal rule-sets were only derived for a pair of animals foraging together, but also because the optimal rules generated by the dynamic games were not necessarily simple to describe, although their salient features could broadly be approximated to a rule-of-thumb [30], [32]. In a thorough exploration of the dynamic game [30], it was demonstrated that changes in the overall behaviours and energetic gains by individual group members weren't necessarily predictable relative to changes in the initial parameters of the system. This suggests that there may be a confounding relationship between the finer, non-predictable components of the optimal rule-sets and the outputs of these models. These unpredictable relationships might be removed if simpler, rule-of-thumb approximations of the optimal rule-sets are followed, such as those explored in [33], [34]. Here, we therefore run some very simple individual-based simulations where pairs of individuals follow the type of rule-of-thumb described by Rands *et al.* [30], [32], to explore whether the level of complexity seen in [30] is due to the finer details of the true optimal rule-sets, or whether a much simpler rule-set will generate a similar level of complexity in the behaviour of a foraging pair.

## Methods

### Model outline

Each simulation followed the changes in state of a pair of individuals over a series of consecutive periods of time. The state of an individual *i* at time *t* was characterised by *x_i_*(*t*), a level of energy reserves which could fall anywhere between 0 and *x_max_*. At the beginning of a period, an individual *i* could choose to either forage or rest, based upon its energetic reserves. If it **RESTED**, it was assumed to use energy such that at time *t*+1 its state was reduced by a randomly selected amount *k_t_* drawn from a normal distribution with mean loss κ*_i_* and standard deviation σ*_i_* (bounded such that if any random loss generated was less than zero, a zero value was returned). If it **FORAGED**, it also incurred a loss *k_t_* with mean κ*_i_* and standard deviation σ*_i_*, but then increased its state by a randomly selected amount *g_t_* drawn from a normal distribution with mean gain γ*_i_* and standard deviation ρ*_i_* (again, where any randomly generated gain less than zero was reported as a zero value). In summary, if an individual with state *x_i_*(*t*) at time *t* rested, its state at time (*t*+1) was *x_i_*(*t*) − *k_t_*. If it foraged, its state at time *t*+1 was *x_i_*(*t*) − *k_t_*+*g_t_*.

However, if *x_i_*(*t*+1) fell at or below 0, the individual was assumed to have starved, and its state remained at 0 for all subsequent periods. If foraging pushed the individual's state above *x_max_*, it was assumed that *x_i_*(*t*+1) = *x_max_*.

**Figure 1:**
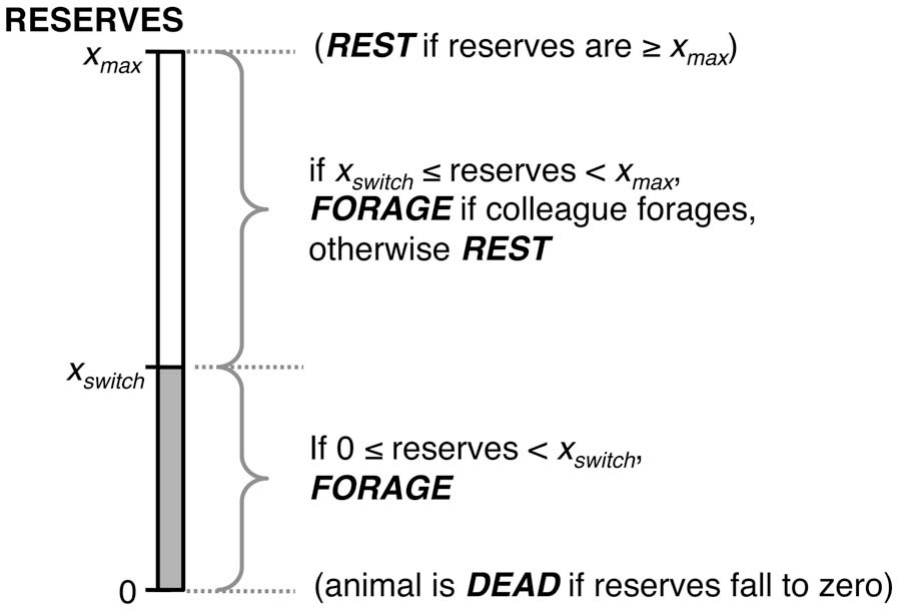
Sketch of an individual's rule set. This is the rule set used by an individual when paying attention to both its own state, and that of its neighbour.

At each timestep, the behaviour of each individual was determined by a rule-of-thumb that depended upon the value of its own state and that of its colleague (sketched in Figure 1). Following the rule-set described proposed by Rands *et al.* [30], [32], each individual *i* had a switchpoint *s_i_*, bounded between 0 and *x_max_*. If its state at time *t* fell below this switchpoint, it foraged during the period (in order to avoid starvation). If its state at time *t* fell at or above this switchpoint, it rested unless its colleague *j* was foraging (determined by whether the colleague's state *x_j_*(*t*) was below the colleague's switchpoint *s_j_*), in which case individual *i* foraged. Therefore, the actions of the pair were determined by the individual that was most at risk of starvation [32]. If an individual's colleague had starved, it foraged if its reserves fell below *s_i_*, and rested otherwise. Summary statistics were collected as described below.

As well as considering pairs of individuals following behavioural rules that paid attention to the state of each other at a given moment in time (referred to as a ‘**paired**’ rule), a set of simulations with identical parameters to the above were conducted, but where both individuals within a pair followed a ‘**solo**’ rule. Here, they foraged if their reserves fell below their own switchpoint, and rested otherwise (identical to the rule described above if an individual's colleague had starved).

### Parameterising identical pair members

For the cases where both pair members were considered to be identical in their metabolic requirements and expenditures, model exploration was conducted by generating 5,000 randomised sets of the five parameters, where parameters were randomly drawn from the following ranges: γ*_i_*: [0, 3]; ρ*_i_*: [0,2]; κ*_i_*: [0, γ*_i_*]; σ*_i_*: [0, 2]; and *s_i_*: [0, *x_max_*] (assuming that *x_max_* = 100 for both members of a pair). Holding the other four parameters constant, each parameter in turn was systematically altered in 24 equal steps across the following ranges (which doubled the ranges that fixed parameters were drawn from): γ*_i_*: [1/4, 6]; ρ*_i_*: [1/6,4]; κ*_i_*: [γ*_i_*/12, 2γ*_i_*]; σ*_i_*: [1/6, 4]; and *s_i_*: [4, 96]. A full set of simulations was run for each of the 24 alterations. Each set of simulations followed 10,000 pairs of individuals over 2,500 timesteps, where each individual was independently allocated a state drawn for a uniform distribution between 0 and *x_max_* at the first timestep.

### Parameterising non-identical pair members

For the cases where differences were considered between the members of a pair, the five parameters used by individual A were first randomly drawn from similar ranges to above, using: γ*_a_*: [0, 3]; ρ*_a_*: [0,2]; κ*_a_*: [0, γ*_a_*]; σ*_a_*: [0, 2]; and *s_a_*: [0, *x_max_*]. The parameters for individual B were assumed to be identical to those of individual A, apart from one single parameter which was systematically altered in 24 steps over the following ranges: γ*_b_*: [γ*_a_*/24, γ*_a_*]; ρ*_b_*: [ρ*_a_*/24, ρ*_a_*]; κ*_b_*: [κ*_a_*/24, κ*_a_*]; σ*_b_*: [σ*_a_*/24, σ*_a_*]; and *s_b_*: [*s_a_*/24, *s_a_*]. During the systematic alteration of each single parameter, the other four parameters were held constant, and 5,000 sets of constant parameters were considered. Again, each set of simulations followed 10,000 pairs of individuals over 2,500 timesteps, where each individual was independently allocated a state drawn for a uniform distribution between 0 and *x_max_* at the first timestep.

### Data collected

The proportion of simulations where both members of the pair survived was recorded. If both pair members survived through to the end of a simulation, the mean difference in energy reserves was calculated by taking the mean absolute difference in energy reserves between the pair members across all 2,500 of the timesteps simulated. From these pair means, a population mean difference in state was then calculated. Similarly, the identity of the individual within a surviving pair that was heaviest was tracked throughout the 2,500 steps of the simulation, and the number of times that the identity changed was calculated. The mean number of times that switching occurred was calculated across all the surviving pairs for each set of simulations.

Using these criteria, the proportion of pairs surviving, the mean energetic differences within the pairs, and the mean number of switches in heaviness were calculated for pairs following the ‘paired’ behavioural rule and the ‘solo’ behavioural rule. Also, the number of times each individual foraged or rested, and the number of times the pairs foraged together, rested alone, or conducted differing behaviours was calculated, and the proportion of the time they were doing the same thing (their ‘synchronisation’) was also recorded.

All simulations were conducted using code written to double-point precision within *C*++. Output data were visualised using *R* 2.11.1 [35], with the aid of *R Commander* 1.5–6 [36].

### Comparison to earlier results

Although very similar in final output to the current exploration, the dynamic game described in detail by Rands *et al*. [30] was parameterised differently to the current simulations. Rather than an overall metabolic cost regardless of activity which was countered by energetic gain when foraging (as assumed in the current model), the dynamic game considered potentially different metabolic costs of foraging and of resting, in addition to the energetic gain whilst foraging. The model presented here therefore uses a simplification of the costs outlined in the dynamic game. The energetic cost framed here should be directly compared to the resting cost framed in [30]: the foraging cost in the dynamic game has no direct comparison, as this has been absorbed into the net gain that occurs in the current model. The switchpoint considered here was not considered in [30]. In the game results, synchronisation was calculated differently to the way it was quantified here, and so the results presented here are compared with the changes in the *C* and *S* statistics of synchronisation initially proposed in [37], that were used to characterise synchronisation in the dynamic game (and were argued to be more accurate than the ‘synchronisation’ coefficient *D′* that was also presented in [30]).

Note that the results reported in [30]'s Table 2 that are repeated here may not appear identical to those in the original version, as trends were calculated in a different manner. To correct for this, the following changes were made. For the non-identical results concerning energetic gain, ‘player one’ in [30] corresponds to the current individual B, whilst for the non-identical results concerning energetic loss, ‘player one’ in [30] corresponds to the current individual A. Furthermore, the results presented for energetic gain in [30] represents what happens when the difference in parameter values between the pair members is increased (rather than decreased, as discussed here), and therefore the trends for energetic gain described in [30] have been reversed for their presentation here.

## Results

Tables 1 and 2 describe the trends for all the parameter manipulations examined. Figures illustrating these trends are given in the Supporting Information.

**Table 1:** Effects of model parameters on measures of identical and non-identical foraging pairs.

	IDENTICAL FORAGERS							NON-IDENTICAL FORAGERS						
	direction of change in property in response to increase in:					comparative results from [30]		direction of change in property in response to increase in:					comparative results from [30]	
	gain		cost					gain		cost				
PROPERTY	mean	s.d.	mean	s.d.	switchpoint	gain	cost	mean	s.d.	mean	s.d.	switchpoint	gain	cost
**proportion of population where both members of a pair are alive after 2,500timesteps** (see also Figure S1)														
using ‘paired’ rule	↑↑	↑↑	↓↓	↓↓	↑↑			↑↑	↑↑	↓↓	↓	↑↑		
using ‘solo’ rule	↑↑	↑↑	↓↓	↓↓	↑↑			↑↑	↑↑	↓↓	↓	↑↑		
larger value	pair	pair	pair	pair	pair			pair	pair	pair	pair	pair		
**mean number of times pair members switch between being lightest and heaviest** (see also Figure S2)														
using ‘paired’ rule	∩∩	↑↑	∩∩	↑↑	↑↑			↑	↑	↑↑	↑↑	∪∪		
using ‘solo’ rule	∩∩	↓↓	∩∩	∩∩	↓↓			↑↑	↓↓	∩∩	∩	↑↑		
larger value	solo	solo	solo	solo	solo			solo	solo	solo	solo	shift		
**mean difference in energetic reserves between pair members throughout simulation** (see also Figure S3)														
using ‘paired’ rule	↓↓	×	∪∪	↑	∩∩	↓↓	↑↑	∩∩	∩	↓↓	↓	∩∩	×	↓
using ‘solo’ rule	∪∪	×	∪∪	↑↑	∪∪			∩	×	∪∪	∪	↓↓		
larger value	pair	pair	pair	pair	pair			pair	pair	pair	pair	shift		
**mean number of times player A switches behaviour** (see also Figure S4a)														
using ‘paired’ rule	∩∩	↓↓	∩∩	↓↓	∪∪	↓↓	↑↑	×	↓↓	↑	×	×	×	↓
using ‘solo’ rule	∩∩	↓↓	∩∩	↓↓	↓↓			∩∩	↓↓	×	×	∩∩		
larger value	pair	pair	shift	pair	pair			shift	pair	pair	pair	pair		
**mean number of times player B switches behaviour** (see also Figure S4b)														
using ‘paired’ rule	identical to player A							↑↑	↓	∩∩	∩∩	×	×	↓↓
using ‘solo’ rule								↑↑	↓↓	∩∩	∩∩	∩∩		
larger value								pair	shift	pair	shift	pair		

Details refer to the direction of change seen when the value of a given parameter was increased. In the case of the non-identical simulations, the parameter value for individual A was fixed, and that of the focal individual B was assumed to increase from a low value upwards to eventually equal that of individual A. ‘↑↑’ indicates a strict increase in the statistics measured relative to the increase in the altered parameter, ‘↓↓’ indicates a strict decrease, ‘∪∪’ indicates a strict intermediate minimum, ‘∩∩’ indicates a strict intermediate maximum, and ‘×’ indicates no simple pattern. Single characters (‘↑’, ‘↓’, ‘∪’ and ‘∩’) indicate similar trends, but with more noise in the results explored. In comparing the effects of paired or solo foraging to identify which of these yielded the larger value, ‘pair’ means that the paired foragers had the higher values, ‘solo’ meant the unpaired foragers had the highest values, and ‘shift’ indicates that there was a change between paired and unpaired individuals having the higher values as the parameter itself changed. See the Methods section for information about how the comparative results from [30] are presented here.

**Table 2:** Effects of model parameters on behaviours of identical and non-identical foraging pairs.

	IDENTICAL FORAGERS							NON-IDENTICAL FORAGERS						
	direction of change in property in response to increase in:					comparative results from [30]		direction of change in property in response to increase in:					comparative results from [30]	
	gain		cost					gain		cost				
PROPERTY	mean	s.d.	mean	s.d.	switchpoint	gain	cost	mean	s.d.	mean	s.d.	switchpoint	gain	cost
**proportion of times player A forages** (see also Figure S5a)														
using ‘paired’ rule	↓↓	↓↓	↑↑	↑↑	↑↑	↓↓	↑↑	↑	×	×	×	×	↑↑	↑↑
using ‘solo’ rule	↓↓	↓↓	↑↑	↑↑	↑↑			↑	∪∪	×	×	↑↑		
larger value	pair	pair	pair	pair	pair			pair	pair	pair	pair	pair		
**proportion of times player B forages** (see also Figure S5b)														
using ‘paired’ rule	identical to player A							∩∩	∪∪	↑↑	↑↑	∪∪	↑↑	↑↑
using ‘solo’ rule								∩	×	↑↑	↑↑	↑↑		
larger value								pair	pair	pair	pair	pair		
**proportion of time players are synchronised** (see also Figure S6)														
using ‘paired’ rule	↑↑	↓↓	∪∪	↓↓	↓↓	↓↓	↑↑	∪	×	↑↑	↑↑	∩∩	↑↑	↑↑
using ‘solo’ rule	∪∪	∪∪	∪∪	∪∪	∪∪			×	×	↑↑	↑↑	∪∪		
larger value	pair	pair	pair	pair	pair			pair	pair	pair	pair	pair		
**proportion of time players forage together** (see also Figure S7a)														
using ‘paired’ rule	↓↓	↓↓	↑↑	↑↑	∩∩	↓↓	↑↑	∩	×	↑↑	↑↑	↑↑	↑↑	↑↑
using ‘solo’ rule	↓↓	↓↓	↑↑	↑↑	↑↑			∩	×	↑↑	↑↑	↑↑		
larger value	pair	pair	pair	pair	pair			pair	pair	pair	pair	pair		
**proportion of time players rest together** (see also Figure S7b)														
using ‘paired’ rule	↑↑	↑↑	↓↓	↓↓	↓↓	↑↑	↓↓	∪∪	×	↓	×	×	↓↓	↓↓
using ‘solo’ rule	↑↑	↑↑	↓↓	↓↓	↓↓			×	×	↓↓	↓↓	↓↓		
larger value	pair	pair	pair	pair	pair			pair	pair	pair	pair	pair		
**proportion of time player A forages and player B rests** (see also Figure S7c)														
using ‘paired’ rule	↓↓	↑↑	∩∩	↑↑	↑↑	↓	×	×	×	↓↓	↓↓	↓↓	↑↑	↓↓
using ‘solo’ rule	∩∩	∩∩	∩∩	∩∩	∩∩			∪∪	×	↓↓	↓↓	↓↓		
larger value	solo	solo	solo	solo	solo			solo	solo	solo	solo	solo		
**proportion of time player A rests and player B forages** (see also Figure S7d)														
using ‘paired’ rule	identical to previous							∩	∩	↑	↑	×	↓	↑
using ‘solo’ rule								∩	∩	↑↑	↑↑	↑↑		
larger value								solo	solo	solo	solo	solo		

See the legend to Table 1 for full details of content.

### Identical pair-members

Pair survival was very predictable according to parameter manipulation for both paired and solo foragers: increasing gain or the range of gain seen enhanced survival, and increasing costs led to a fall in survival. Increasing the switchpoint had a similar effect to increasing gain, as the baseline energy reserves of the pair was moved further and further away from the point of starvation. Although the paired individuals were always the most likely to survive (because their behaviour was driving them to avoid starvation by putting on extra reserves), the exact difference in survival between paired and solo individuals modelled using the same parameters did not show a simple increase or decrease, but was largely similar between the two behavioural types (see Figure S1).

The amount of switching in reserves from heaviest to lightest between behaviours was less predictable, with distinct intermediate minima or maxima being seen for both mean gain and mean cost, but increasing the variation of these terms had some effect on switching between heaviest and lightest (Table 1, Figure S2). Increasing the behavioural switchpoint caused paired individuals to increase the number of times they swapped over, but reduced the times that solo individuals changed. In all cases, it was the solo individuals that swapped their position more often, which makes sense – both individuals should be near their behavioural switchpoint most of the time, and should therefore swap more often than if one individual is heavier than the other. For further discussion, see [30], [32]. Similarly, the mean difference in the energy reserves between the pair members tended to be unpredictable (Table 1, Figure S3), in contrast to the clear trends given by the game predictions in [30]. Switching behaviours also showed similarly indistinct trends, in contrast to the results from [30] (Table 1, Figure S4).

The actual behaviours shown by individuals showed similar trends to [30], with a distinct reduction in time spent foraging if gain (or variance in gain) fell, and a distinct increase in foraging if energy expenditure or its variance increased (Table 2, Figure S5). Increasing the switchpoint value led to an increase in foraging behaviour as well. Paired individuals tended to forage slightly more often (evident in the black lines appearing above the red lines in Figure S5), again being driven by either their own or their colleague's proximity to the switchpoint value.

The proportions of times individuals foraged together or rested together showed similar trends to what individuals tended to be doing, and this is reflected in the synchrony changes of paired individuals, but not in the synchrony changes of individuals following solo rules (Table 2, Figure S6). It may therefore seem unusual that the asynchronous activities shown by paired individuals followed any sort of trend, but there were changes that echoed those of the proportion of times pairs foraged together (Table 2, Figure S7). Solo individuals again didn't coordinate their activities, and therefore didn't show any predictable trends. In general, the paired behaviours of individuals in this simulation followed the game results given in [30].

### Non-identical pair-members

The effects of changing parameter values on the survival of non-identical pairs had similar effects to identical pairs. There was more predictability in the switches of individuals between being heaviest and lightest, which tended to increase with increasing gains or costs. The mean difference in energetic reserves between pair members did not yield many obvious relationships, although reducing the difference between the energetic costs of the two individuals did mean that their energetic reserves differed less, as would be expected. These patterns (or lack of them) reflected the game results in [30].

The patterns of behaviour shown by both single individuals and by pairs yielded very few trends when energetic gains and variation in gain were considered, which does not reflect the clear-cut results in [30]. Increasing costs yielded much more obvious quantifiable changes, and most of these followed the patterns given in [30]. Changing the switchpoint values tells us little about the behaviours expected between non-identical pair members.

## Discussion

The simulations conducted here used a rule-of-thumb approximation of the rules described by Rands *et al*. [30], [32]. These original rule-sets, calculated as optimal solutions to evolutionary dynamic games, were more complex than the rules-of-thumb described here, and yielded complex changes in results that were difficult to relate to simple changes in the parameters that the foragers were faced with [30]. Here, we demonstrate that even if the rules of interaction between the pair members are reduced to a much more straightforward form than that suggested by the optimal policies of the dynamic game, the combined behaviours of the pair are still difficult (or impossible) to predict without having to simulate their behaviour given a particular parameter set. This lack of obvious relationships is especially true where non-identical pairs of foragers are considered. Some of the results shown here agree with those given by the dynamic game model [30] – in particular, the behavioural proportions of individuals and pairs for both identical and non-identical pair members tended to follow game predictions, but there was much less agreement when considering energetic gain in non-identical individuals, or the number of times individuals tended to switch from one behaviour to the other.

We should expect that using two differing modelling techniques with different assumptions is likely to yield differing results. The original dynamic game models presented in [30], [32] are based on a few simple biologically-motivated assumptions, but it is not possible to predict the exact form of the optimal rule sets they generate for a given set of parameters without having to calculate them computationally. Here, I have demonstrated that creating a rule-of-thumb approximation of these optimal rules that would be simpler to implement yields results that are just as difficult to relate to the initial parameterisation of the system. Which of these two techniques is the most biologically relevant is debatable. The former game theoretic technique is based on a simple set of ecologically-grounded assumptions, but yield policy sets which are heavily reliant on recognising subtle physiological differences in both self and foraging partner. Although the latter rule-of-thumb simulation technique is much less reliant on an individual having an exact knowledge of the energetic state of both itself and its partner, but the assumptions behind the rules used are arguably not shaped by the effects of the social and physical environment on the fitness of individuals, but are rather guessed by the modeller as being ‘good enough’ rules (arguably in much the same way as many of the rule-sets that go into simulations of the collective behaviours of individuals [31]). This suggests that using estimated rules-of-thumb should by less justifiable if we intend to capture biologically-appropriate behaviour within a model. However, from a modelling perspective, rules-of-thumb are much more straight-forward to both parameterise and implement within simulations, and therefore the exploration of the differences between the outputs of the two approaches conducted here is necessary to identify where possible differences could lie.

Sumpter [38] also presents a model based on [32], exploring social foraging by a pair of animals that can choose to forage or rest during a given period. The model describes a cost-benefit analysis, where foraging leads to a state-dependent increase in fitness that is balanced by a fitness reduction due to a constant predation cost. The severity of this cost is reduced if the pair of individuals choose to forage at the same time. Resting is seen as a neutral activity, which incurs no additional gains or reductions in fitness. The model therefore frames a scenario where an individual's behaviour is not tempered by a possible risk of starvation, and also where predation doesn't lead to a potential cataclysmic reduction in future fitness to zero (which could occur if the individual was eaten). Despite these major differences, Sumpter's model gives a similar strategy to the rule-of-thumb suggested in [30], [32] that is implemented in results described in the current paper, which could justify tying the purely mechanistic rule-of-thumb with a more solid functional grounding. Sumpter also extends this single-decision framework to consider a co-ordination game where resting does incur a cost, and where there is some stochasticity in metabolic expenditure. Stochasticity appears to be implemented in a different manner to that used here and in [30], [32], which may explain why pairs of individuals within a pair in [38] are never able to switch in role from being the heavier to the lighter individual. Therefore, in Sumpter's model, the nutritional state of the members of a pair will not be a consequence of synchronisation, whilst (contrary to Sumpter's discussion), in the dynamic games of Rands *et al.* [30], [32], synchronisation is a direct result of the assumptions made about how changes in energetic state are related to fitness.

What I demonstrate in this manuscript is a lack of agreement between two modelling techniques (the dynamic games presented in [30], [32], and the rule-of-thumb simulations presented here), which suggests that the rules-of-thumb extracted from the dynamic games may not be catching some of the subtler behavioural interactions between the pair members that are specified by the games' more detailed behavioural policies. However, there is agreement for some of the parameters, which in turn suggests that the simplified rules aren't necessarily inappropriate. Care therefore needs to be taken in translating the subtler details of the dynamic game policies to a format that is computationally simple enough to be implemented usefully within a simulation involving multiple independent agents: it makes more sense to use rules based on some biological reality rather than rules that are based purely on guesswork [31]. It is therefore important to conduct investigations such as those presented here, to allow us to understand the limitations of both techniques. As Hutchinson & Gigerenzer [39] explicitly state, optimality modelling provides us with clues for what heuristics might be being used within natural systems, and conducting comparative tests such as those given here give us a handle on what could work and what might not.

The simulations presented here only consider a pair of foragers, but it is reassuring that the predictions coming from both the current simulations and the dynamic game model give broadly similar trends in response to the energetic parameters put into them. It is computationally difficult to extend state-dependent game theoretic modelling to larger groups (especially if individuals are not identical), and having some validation of these simpler rules gives some support to the generalised multi-agent simulations conducted by Rands *et al*. [33], [34]. There are still many variations on this foraging rule-set to consider theoretically, such as using them to explore how group behaviour affects predation risk (*e.g.* [40]–[46]), or to consider the effects of non-egalitarian decision-making within the group (*e.g.* [23], [34], [47], [48]). Being able to build on validated rule-sets should give us some grounding in how agent-based rules could developed from a functional perspective.

It should also be noted that optimal behaviour as predicted by the dynamic game models may not be biologically valid due to developmental and other constraints. By considering simple approximations of these rules, we are instead considering ‘best attempts’ at good behaviours that could be feasibly selected for in an organism that faces biological constraints in its development. McNamara & Houston [49] argue that we need to pay more attention to how behavioural systems are built to deal with these constraints, as the mechanisms generating these behaviours may not be flexible enough to allow the optimal behaviour to be performed. This would give a finite number of possible sets of behaviours, which an organism would have to use when responding to the environment. Rules that have been generated by natural selection may work very badly [50], whilst some rules that work very well may make little sense [51]. Considering both function and possible mechanistic constraints when creating a model is an important step towards understanding the design of decision-making systems (see, for example, [52]–[56]). Characterising the limitations and differences between both, using techniques such as those described here, is an important step towards this understanding.

## Supporting Information

Figure S1 **Proportions of the population surviving 2,500 timesteps, in response to the manipulation of target parameters.** The left column illustrates the cases where pair members are identical in all their parameters, and the right hand column illustrates the case where the target parameter of individual B is manipulated whilst that of individual A is held constant. From top to bottom, the figures illustrate what happens when the target parameter being manipulated is: the mean energetic gain; the standard deviation of the energetic gain; the mean energetic loss during a timestep; the standard deviation of the energetic loss; and the behavioural switchpoint determining the behaviour shown by an individual dependent upon its energetic reserves. The black line represents the mean proportion alive (± s.d., given as black error bars) when individuals in a pair followed a ‘paired’ rule; the red line represents the mean proportion alive (± s.d., given as orange error bars) when individuals in a pair followed a ‘solo’ rule. Paired and solo lines are displayed slightly offset for clarity. These results are summarised in Table 1.
(PDF)

Figure S2 **Rôle switches in response to the manipulation of target parameters.** Figures display the mean number of timesteps (± s.d.) that individuals switched in their ‘rôle’ between being the heavier and the lighter member of the pair. Layout is as described for Figure S1. These results are summarised in Table 1.
(PDF)

Figure S3 **Mean differences in a pair's energy reserves, in response to the manipulation of target parameters.** Figures display the mean energetic difference (± s.d.) within a pair during a timestep. Layout is as described for Figure S1. These results are summarised in Table 1.
(PDF)

Figure S4 **Changes in individual behavioural switches in response to the manipulation of target parameters.** Figures show: *a*) mean number of behavioural switches (± s.d.) shown by a focal individual (when foragers are identical) or individual A (when foragers are non-identical); *b*) mean number of behavioural switches (± s.d.) shown by individual B (when foragers are non-identical). Layout is as described for Figure S1. These results are summarised in Table 1.
(PDF)

Figure S5 **Changes in individual foraging behaviour in response to the manipulation of target parameters.** Figures show: *a*) mean number of timesteps (± s.d.) where foraging behaviour was conducted by a focal individual (when foragers are identical) or individual A (when foragers are non-identical); *b*) mean number of timesteps (± s.d.) where foraging behaviour was conducted by individual B (when foragers are non-identical). Layout is as described for Figure S1. These results are summarised in Table 2.
(PDF)

Figure S6 **Synchronisation behaviour in response to the manipulation of target parameters.** Figures show: *a*) mean number of timesteps (± s.d.) where both individuals in a pair were conducting the same behaviour. Layout is as described for Figure S1. These results are summarised in Table 2.
(PDF)

Figure S7 **Changes in pair behaviour in response to the manipulation of target parameters.** Figures show: *a*) mean number of timesteps (± s.d.) where both members of a pair foraged; *b*) mean number of timesteps (± s.d.) where both members of a pair rested; *c*) mean number of timesteps (± s.d.) where pair members conducted differing behaviours (when foragers were identical) or where individual A foraged and individual B rested (when foragers are non-identical); *d*) mean number of timesteps where individual A rested and individual B foraged (when foragers were non-identical). Layout is as described for Figure S1. These results are summarised in Table 2.
(PDF)
