# Supplementary figures and images for: Approximating Optimal Behavioural Strategies Down to Rules-of-Thumb: Energy Reserve Changes in Pairs of Social Foragers
Source: PLoS One. 2011 Jul 12;6(7):e22104. doi: 10.1371/journal.pone.0022104 (PMC3134479; doi:10.1371/journal.pone.0022104)

Figure S1

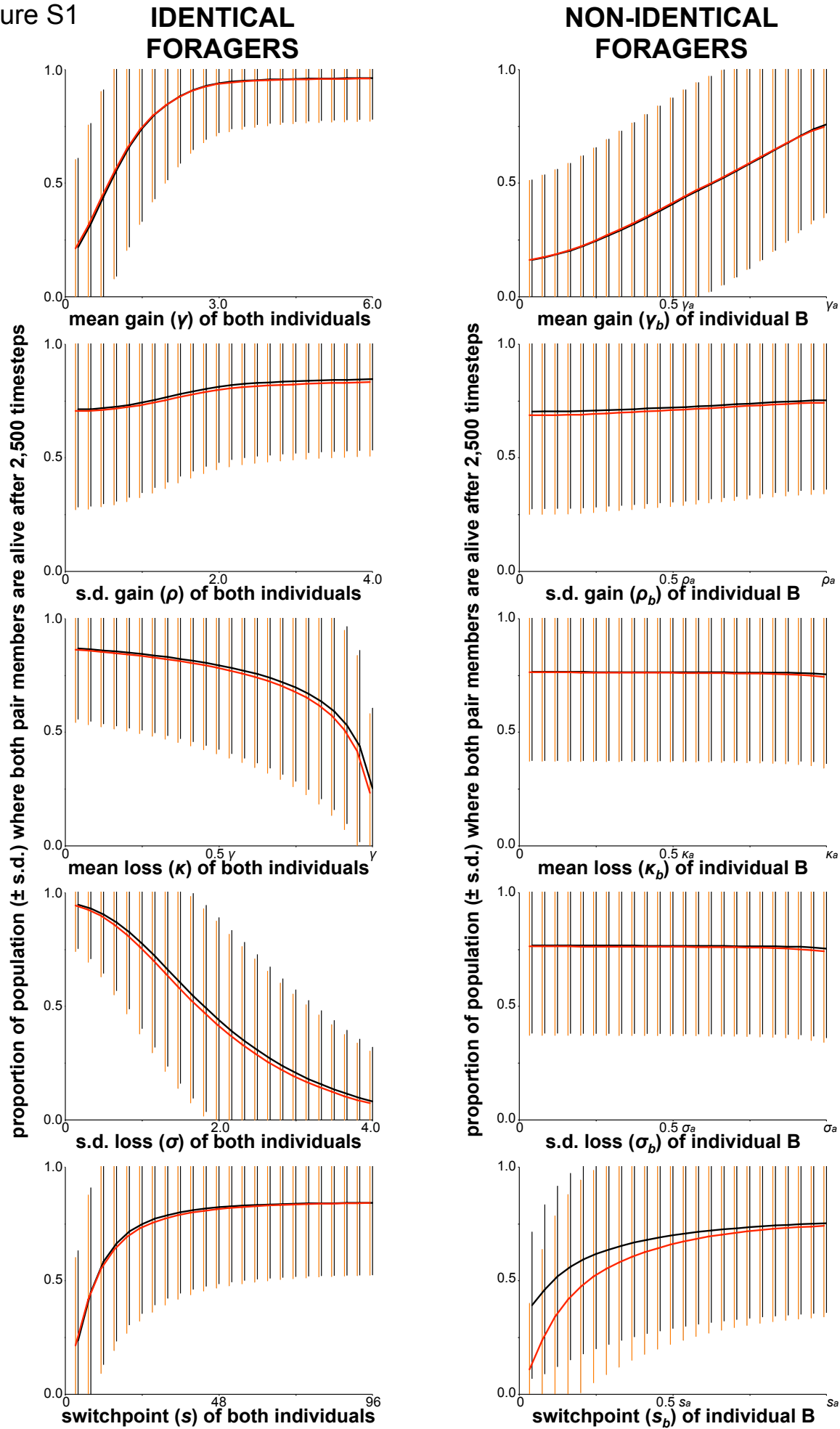

Supplement: Figure S1 — Proportions of the population surviving 2,500 timesteps, in response to the manipulation of target parameters. The left column illustrates the cases where pair members are identical in all their parameters, and the right hand column illustrates the case where the target parameter of individual B is manipulated whilst that of individual A is held constant. From top to bottom, the figures illustrate what happens when the target parameter being manipulated is: the mean energetic gain; the standard deviation of the energetic gain; the mean energetic loss during a timestep; the standard deviation of the energetic loss; and the behavioural switchpoint determining the behaviour shown by an individual dependent upon its energetic reserves. The black line represents the mean proportion alive (± s.d., given as black error bars) when individuals in a pair followed a ‘paired’ rule; the red line represents the mean proportion alive (± s.d., given as orange error bars) when individuals in a pair followed a ‘solo’ rule. Paired and solo lines are displayed slightly offset for clarity. These results are summarised in Table 1. (PDF) [file pone.0022104.s001.pdf]

Figure S2

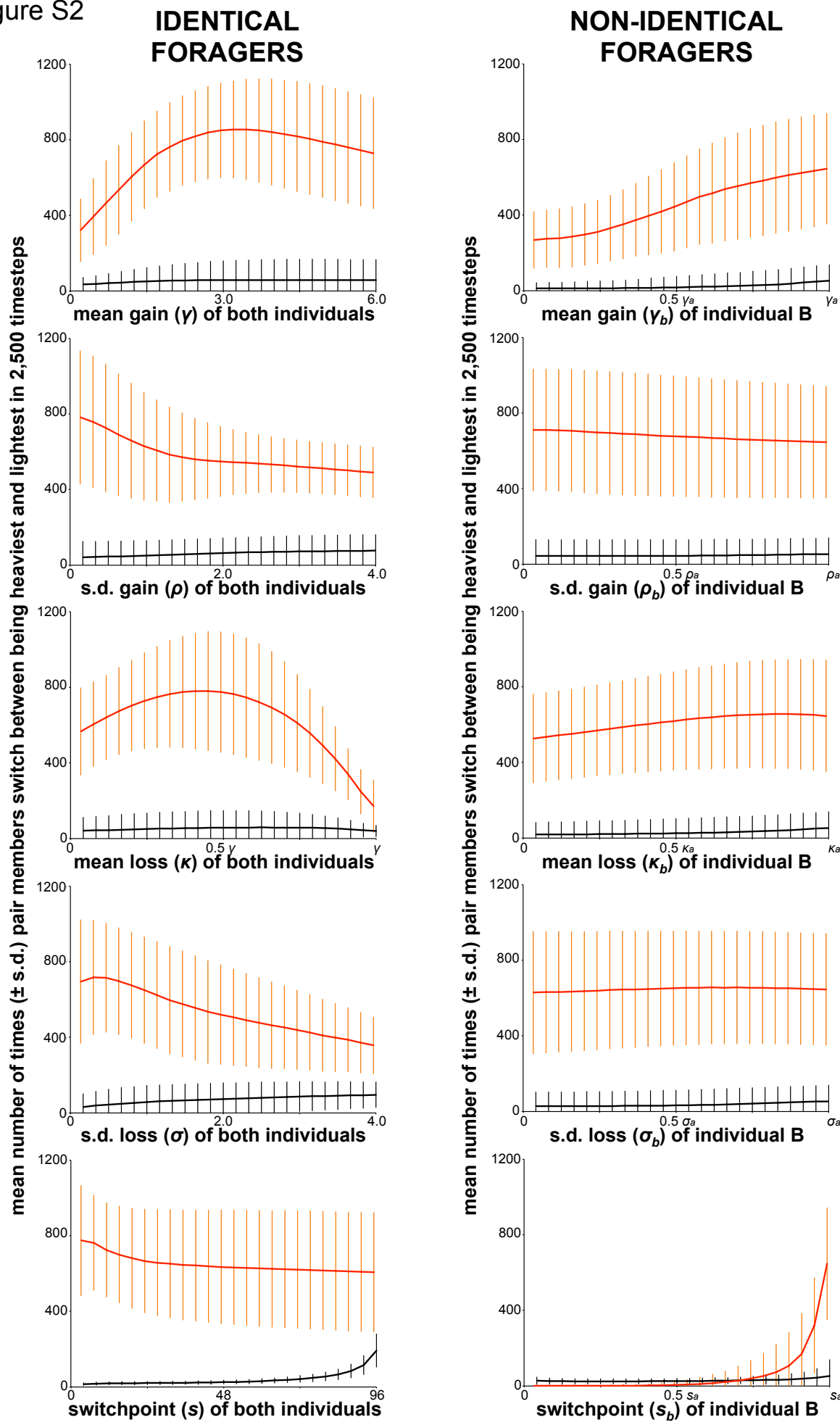

Supplement: Figure S2 — Rôle switches in response to the manipulation of target parameters. Figures display the mean number of timesteps (± s.d.) that individuals switched in their ‘rôle’ between being the heavier and the lighter member of the pair. Layout is as described for Figure S1. These results are summarised in Table 1. (PDF) [file pone.0022104.s002.pdf]

Figure S3

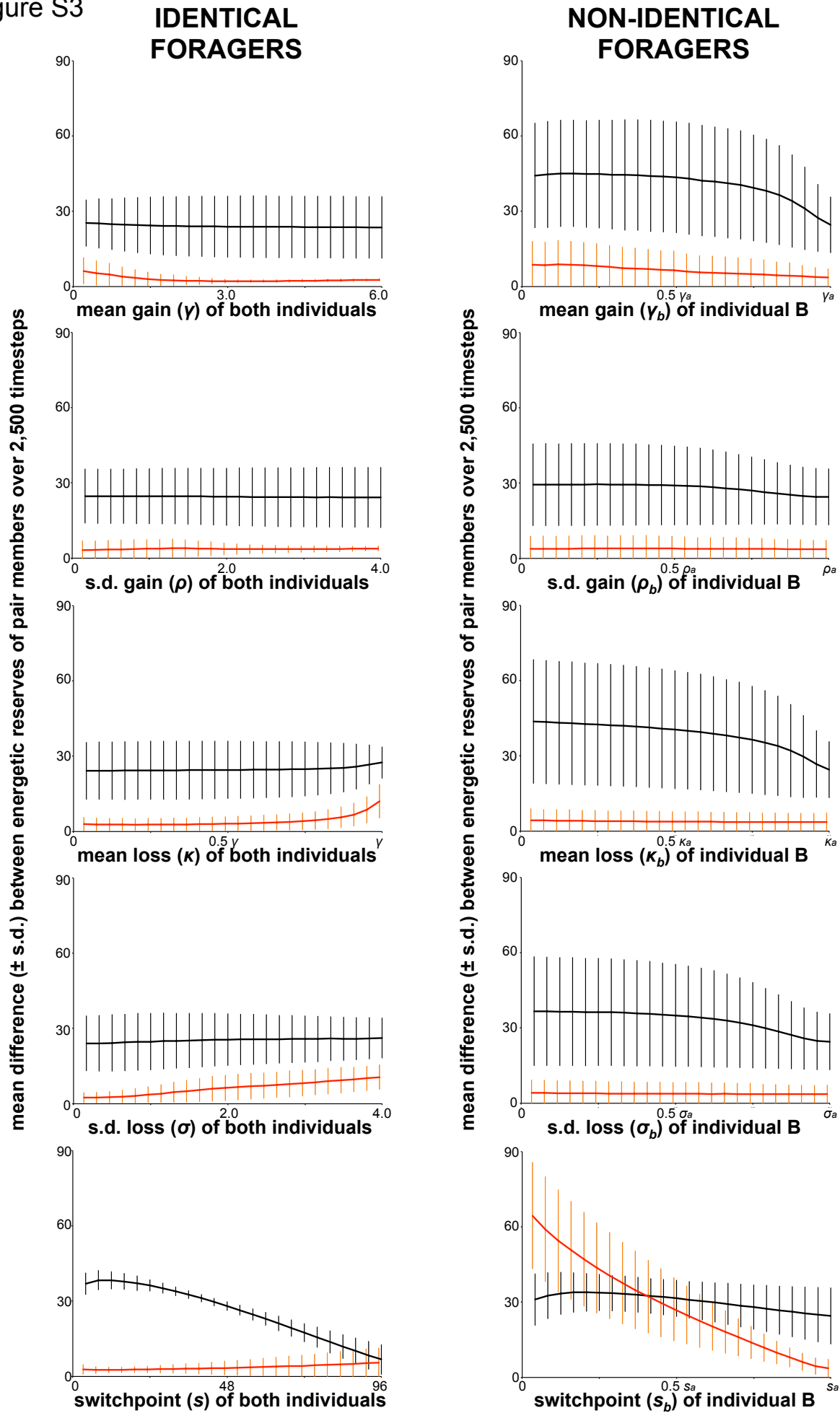

Supplement: Figure S3 — Mean differences in a pair's energy reserves, in response to the manipulation of target parameters. Figures display the mean energetic difference (± s.d.) within a pair during a timestep. Layout is as described for Figure S1. These results are summarised in Table 1. (PDF) [file pone.0022104.s003.pdf]

Figure S6

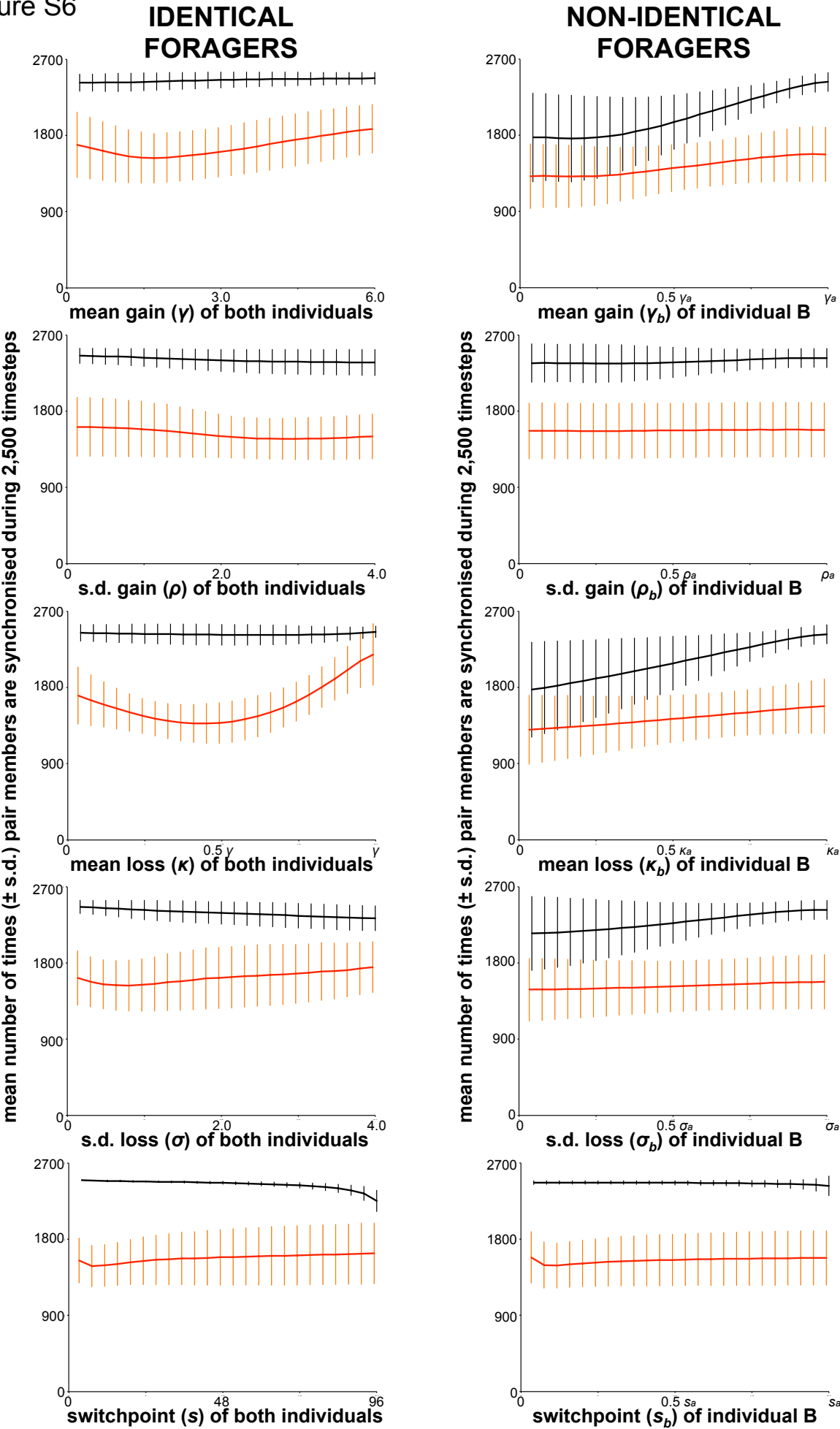

Supplement: Figure S6 — Synchronisation behaviour in response to the manipulation of target parameters. Figures show: a) mean number of timesteps (± s.d.) where both individuals in a pair were conducting the same behaviour. Layout is as described for Figure S1. These results are summarised in Table 2. (PDF) [file pone.0022104.s006.pdf]
