# Supplementary material for: Approximating Optimal Behavioural Strategies Down to Rules-of-Thumb: Energy Reserve Changes in Pairs of Social Foragers
Source: PLoS One. 2011 Jul 12;6(7):e22104. doi: 10.1371/journal.pone.0022104 (PMC3134479; doi:10.1371/journal.pone.0022104)

Figure S4a

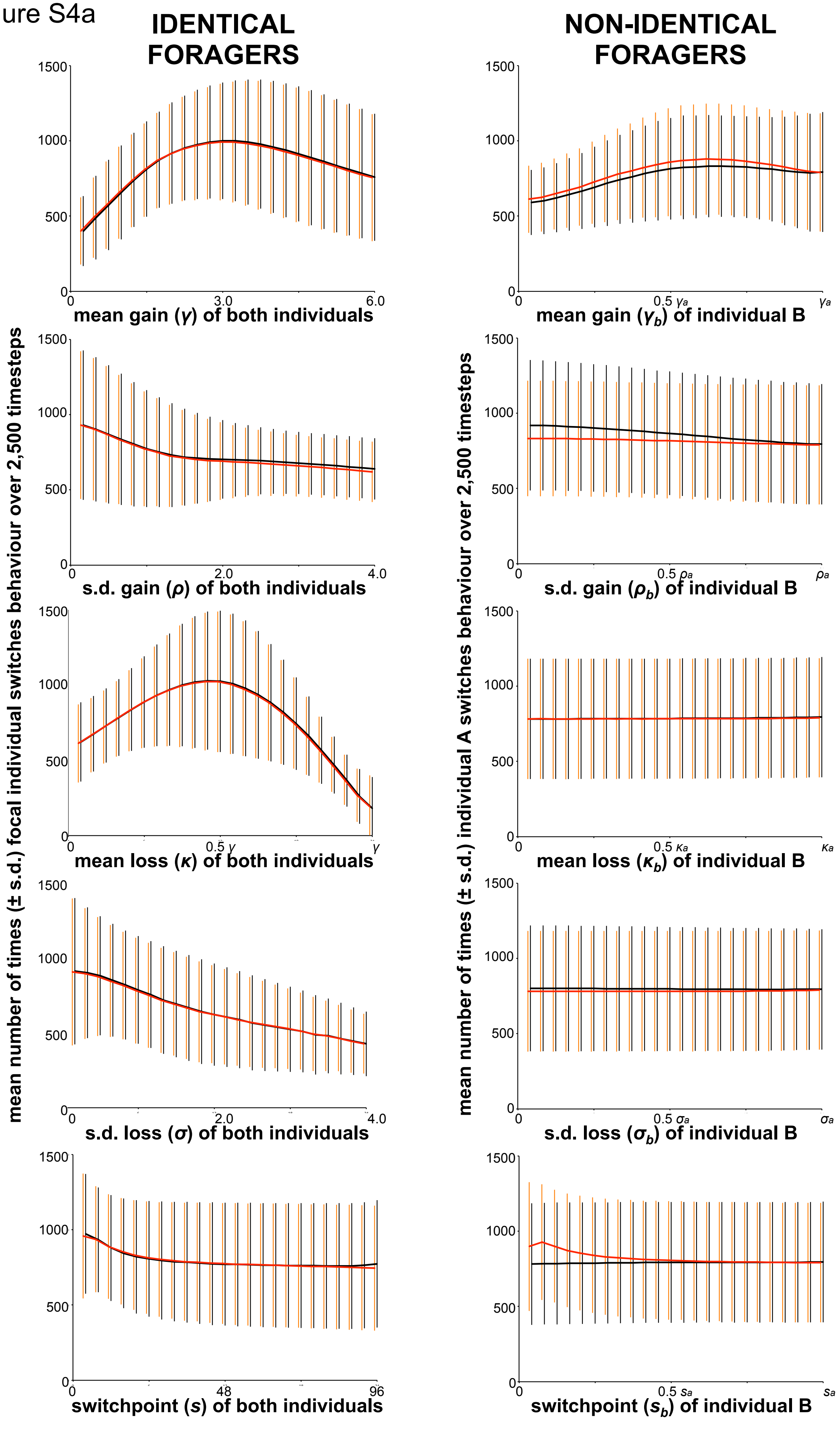
IDENTICAL FORAGERS
NON-IDENTICAL FORAGERS
mean number of times (± s.d.) focal individual switches behaviour over 2,500 timesteps
mean number of times (± s.d.) individual A switches behaviour over 2,500 timesteps
1500
1000
500
0
3.0
6.0
mean gain (γ) of both individuals
0.5 γa
γa
mean gain (γb) of individual B
2.0
4.0
s.d. gain (ρ) of both individuals
0.5 ρa
ρa
s.d. gain (ρb) of individual B
0.5 γ
γ
mean loss (κ) of both individuals
0.5 κa
κa
mean loss (κb) of individual B
s.d. loss (σ) of both individuals
0.5 σa
σa
s.d. loss (σb) of individual B
48
96
switchpoint (s) of both individuals
0.5 sa
sa
switchpoint (sb) of individual B

Figure S4b

IDENTICAL FORAGERS

results for identical foragers are identical to those given in Figure S4a

NON-IDENTICAL FORAGERS

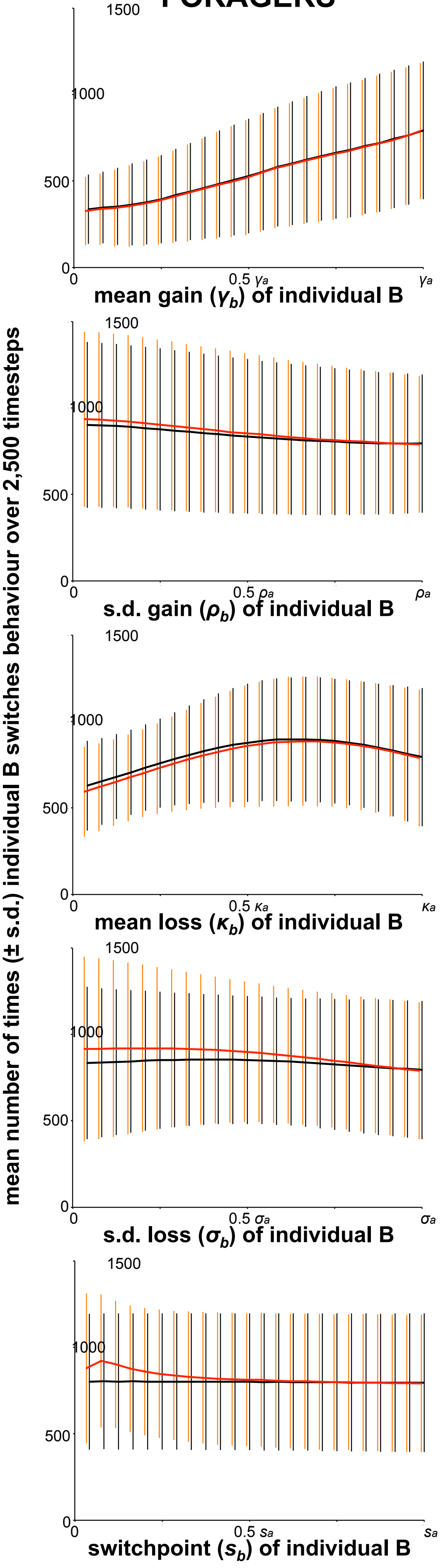

Supplement: Figure S4 — Changes in individual behavioural switches in response to the manipulation of target parameters. Figures show: a) mean number of behavioural switches (± s.d.) shown by a focal individual (when foragers are identical) or individual A (when foragers are non-identical); b) mean number of behavioural switches (± s.d.) shown by individual B (when foragers are non-identical). Layout is as described for Figure S1. These results are summarised in Table 1. (PDF) [file pone.0022104.s004.pdf]
