# Supplementary material for: Approximating Optimal Behavioural Strategies Down to Rules-of-Thumb: Energy Reserve Changes in Pairs of Social Foragers
Source: PLoS One. 2011 Jul 12;6(7):e22104. doi: 10.1371/journal.pone.0022104 (PMC3134479; doi:10.1371/journal.pone.0022104)

Figure S5a

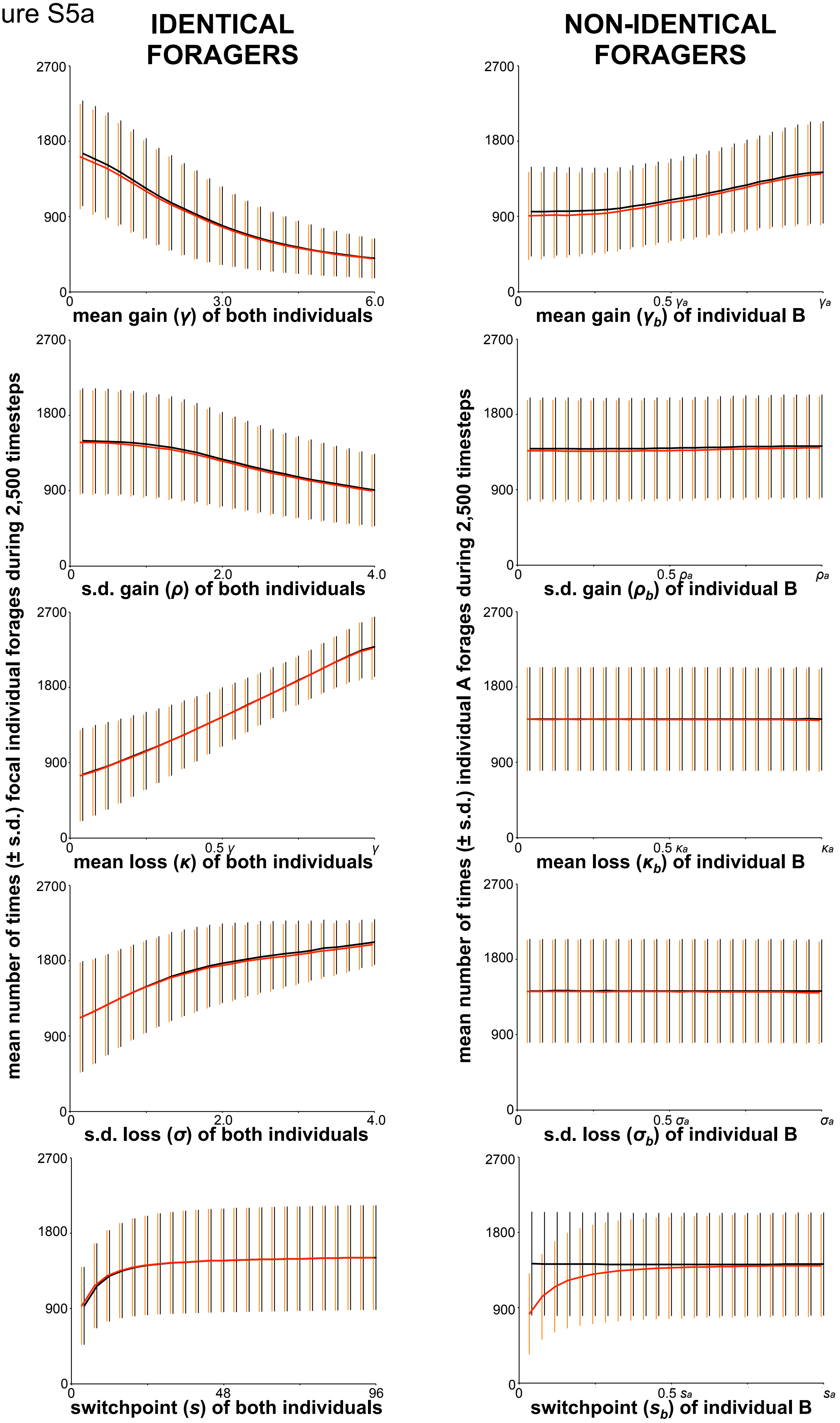
IDENTICAL FORAGERS
NON-IDENTICAL FORAGERS
mean number of times (± s.d.) focal individual forages during 2,500 timesteps
mean number of times (± s.d.) individual A forages during 2,500 timesteps
2700
1800
900
0
mean gain (γ) of both individuals
0
3.0
6.0
mean gain (γ_b) of individual B
0.5 γ_a
γ_a
s.d. gain (ρ) of both individuals
2.0
4.0
s.d. gain (ρ_b) of individual B
0.5 ρ_a
ρ_a
mean loss (κ) of both individuals
0.5 γ
γ
mean loss (κ_b) of individual B
0.5 κ_a
κ_a
s.d. loss (σ) of both individuals
s.d. loss (σ_b) of individual B
0.5 σ_a
σ_a
switchpoint (s) of both individuals
48
96
switchpoint (s_b) of individual B
0.5 s_a
s_a

Figure S5b

IDENTICAL FORAGERS

results for identical foragers
are identical to those given
in Figure S5a

NON-IDENTICAL FORAGERS

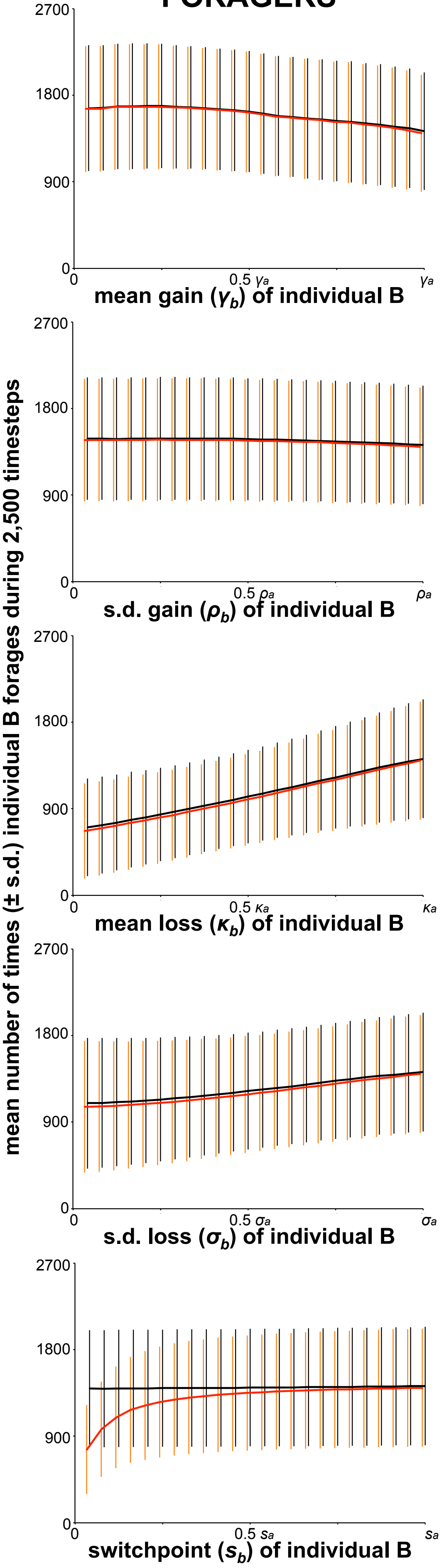

Supplement: Figure S5 — Changes in individual foraging behaviour in response to the manipulation of target parameters. Figures show: a) mean number of timesteps (± s.d.) where foraging behaviour was conducted by a focal individual (when foragers are identical) or individual A (when foragers are non-identical); b) mean number of timesteps (± s.d.) where foraging behaviour was conducted by individual B (when foragers are non-identical). Layout is as described for Figure S1. These results are summarised in Table 2. (PDF) [file pone.0022104.s005.pdf]
