# Supplementary material for: Approximating Optimal Behavioural Strategies Down to Rules-of-Thumb: Energy Reserve Changes in Pairs of Social Foragers
Source: PLoS One. 2011 Jul 12;6(7):e22104. doi: 10.1371/journal.pone.0022104 (PMC3134479; doi:10.1371/journal.pone.0022104)

Figure S7a

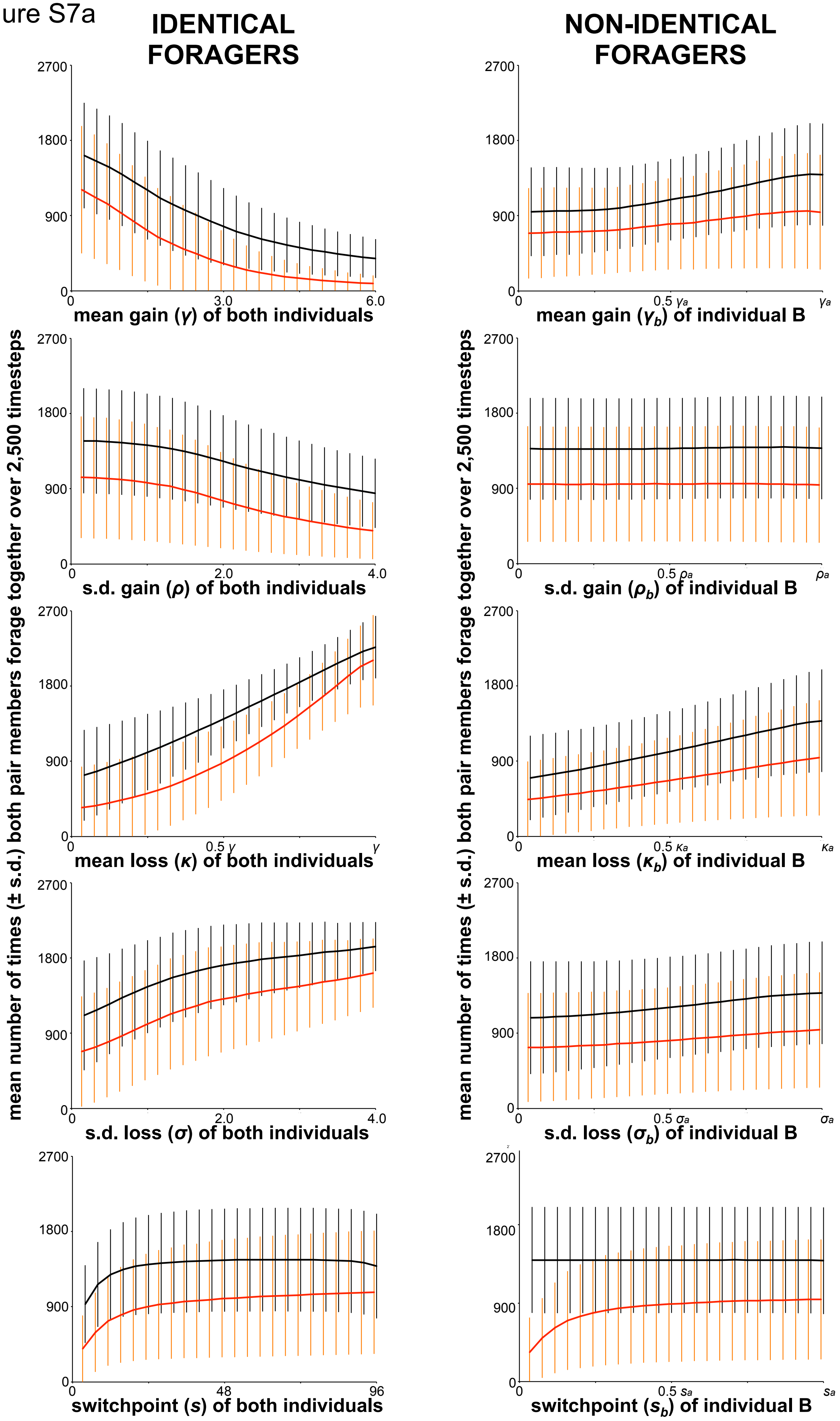
IDENTICAL FORAGERS
NON-IDENTICAL FORAGERS
mean number of times (± s.d.) both pair members forage together over 2,500 timesteps
2700
1800
900
0
3.0
6.0
mean gain (γ) of both individuals
0.5 γa
γa
mean gain (γb) of individual B
2.0
4.0
s.d. gain (ρ) of both individuals
0.5 ρa
ρa
s.d. gain (ρb) of individual B
0.5 γ
γ
mean loss (κ) of both individuals
0.5 κa
κa
mean loss (κb) of individual B
s.d. loss (σ) of both individuals
0.5 σa
σa
s.d. loss (σb) of individual B
48
96
switchpoint (s) of both individuals
0.5 sa
sa
switchpoint (sb) of individual B

Figure S7b

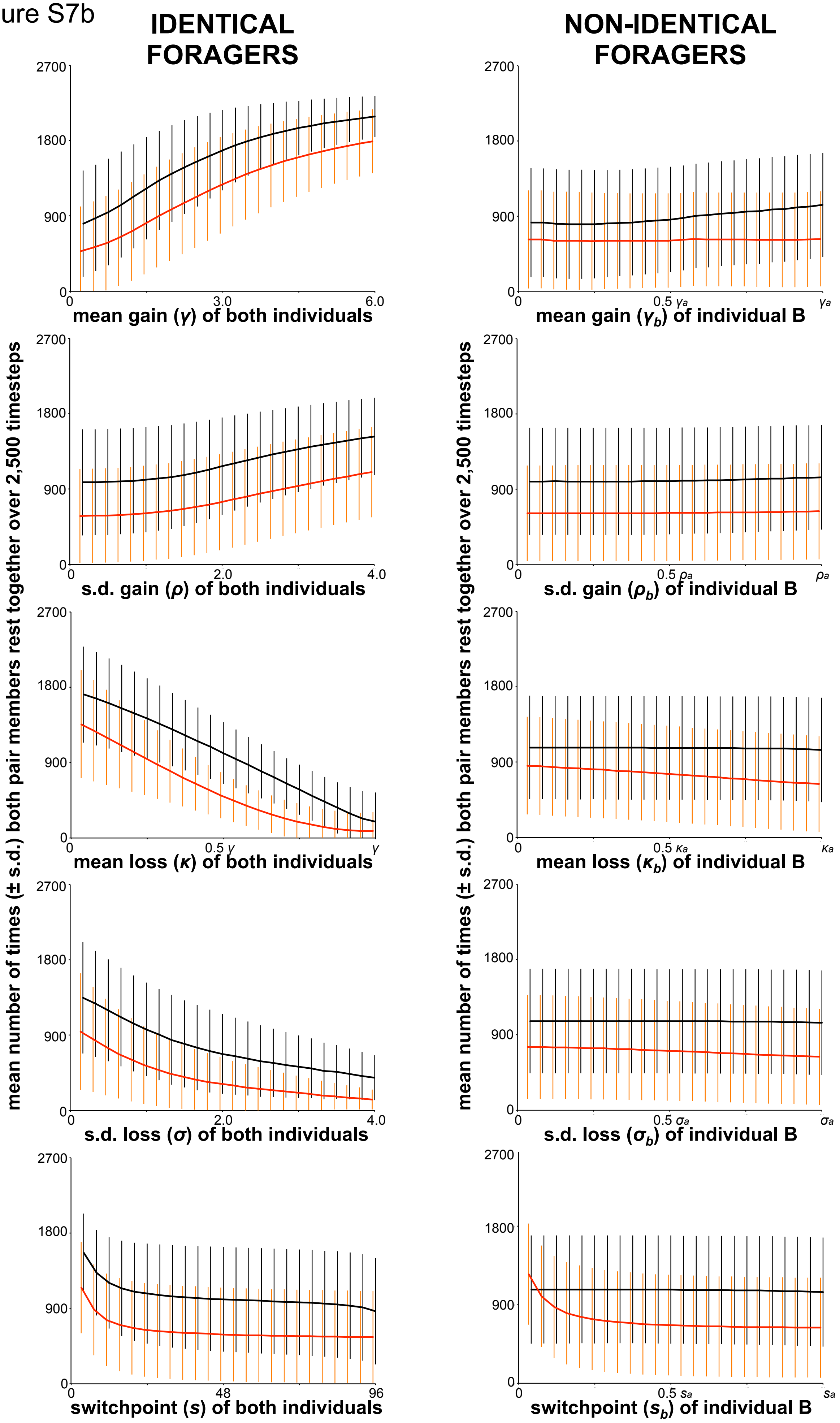
IDENTICAL FORAGERS
NON-IDENTICAL FORAGERS
mean number of times (± s.d.) both pair members rest together over 2,500 timesteps
mean gain (γ) of both individuals
mean gain (γ_b) of individual B
s.d. gain (ρ) of both individuals
s.d. gain (ρ_b) of individual B
mean loss (κ) of both individuals
mean loss (κ_b) of individual B
s.d. loss (σ) of both individuals
s.d. loss (σ_b) of individual B
switchpoint (s) of both individuals
switchpoint (s_b) of individual B
2700
1800
900
0
3.0
6.0
2.0
4.0
48
96
0.5 γa
γa
0.5 ρa
ρa
0.5 κa
κa
0.5 σa
σa
0.5 sa
sa
0.5 γ
γ

Figure S7c

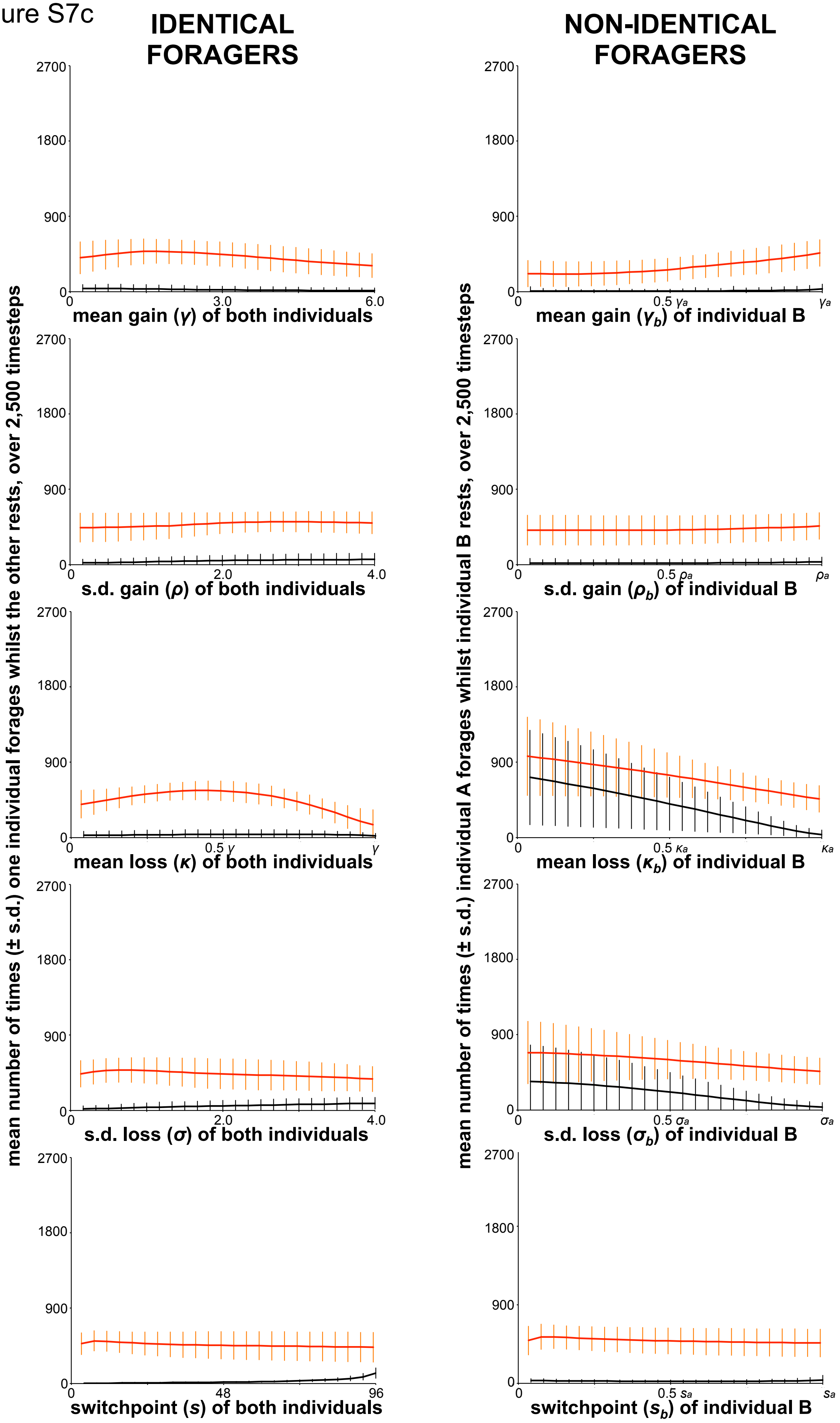

IDENTICAL FORAGERS
NON-IDENTICAL FORAGERS
mean number of times (± s.d.) one individual forages whilst the other rests, over 2,500 timesteps
mean number of times (± s.d.) individual A forages whilst individual B rests, over 2,500 timesteps
2700
1800
900
0
3.0
6.0
0.5 γ_a
γ_a
mean gain (γ) of both individuals
mean gain (γ_b) of individual B
2.0
4.0
0.5 ρ_a
ρ_a
s.d. gain (ρ) of both individuals
s.d. gain (ρ_b) of individual B
0.5 γ
γ
0.5 κ_a
κ_a
mean loss (κ) of both individuals
mean loss (κ_b) of individual B
0.5 σ_a
σ_a
s.d. loss (σ) of both individuals
s.d. loss (σ_b) of individual B
48
96
0.5 s_a
s_a
switchpoint (s) of both individuals
switchpoint (s_b) of individual B

Figure S7d

IDENTICAL FORAGERS

results for identical foragers are identical to those given in Figure S7c

NON-IDENTICAL FORAGERS

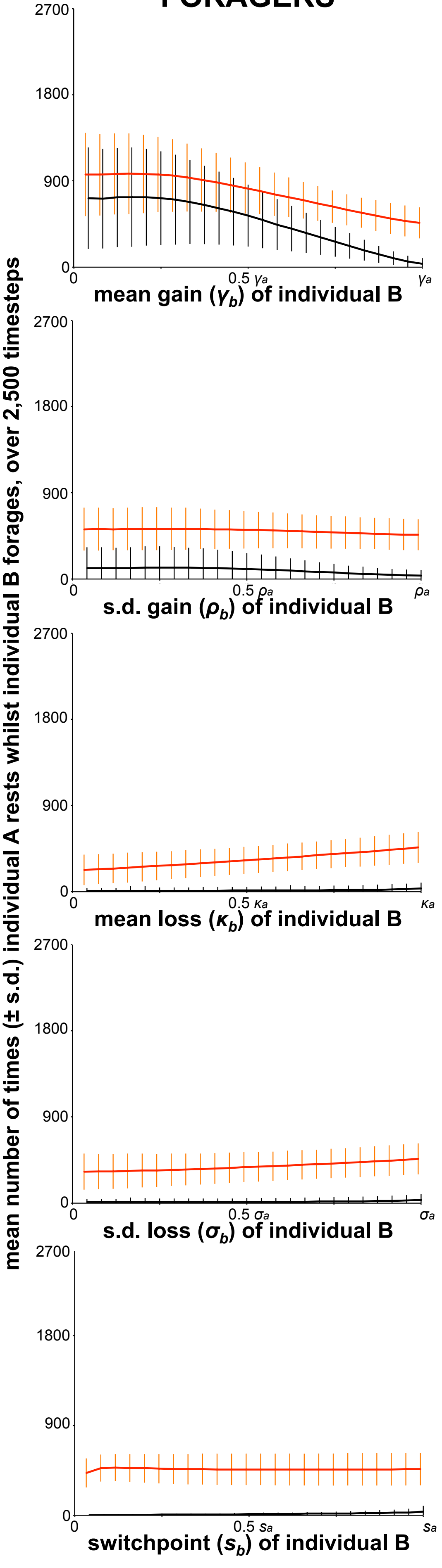

Supplement: Figure S7 — Changes in pair behaviour in response to the manipulation of target parameters. Figures show: a) mean number of timesteps (± s.d.) where both members of a pair foraged; b) mean number of timesteps (± s.d.) where both members of a pair rested; c) mean number of timesteps (± s.d.) where pair members conducted differing behaviours (when foragers were identical) or where individual A foraged and individual B rested (when foragers are non-identical); d) mean number of timesteps where individual A rested and individual B foraged (when foragers were non-identical). Layout is as described for Figure S1. These results are summarised in Table 2. (PDF) [file pone.0022104.s007.pdf]
